# Nanoencapsulated Quercetin Improves Cardioprotection during Hypoxia-Reoxygenation Injury through Preservation of Mitochondrial Function

**DOI:** 10.1155/2019/7683051

**Published:** 2019-06-24

**Authors:** Omar Lozano, Anay Lázaro-Alfaro, Christian Silva-Platas, Yuriana Oropeza-Almazán, Alejandro Torres-Quintanilla, Judith Bernal-Ramírez, Hugo Alves-Figueiredo, Gerardo García-Rivas

**Affiliations:** ^1^Escuela de Medicina y Ciencias de la Salud, Tecnologico de Monterrey, Cátedra de Cardiología y Medicina Vascular, Monterrey, NL 64849, Mexico; ^2^Centro de Investigación Biomédica, Hospital Zambrano-Hellion, San Pedro Garza García 66278, Mexico

## Abstract

The effective delivery of antioxidants to the cells is hindered by their high metabolization rate. In this work, quercetin was encapsulated in poly(lactic-co-glycolic) acid (PLGA) nanoparticles. They were characterized in terms of its physicochemical properties (particle size distribution, *ζ*-potential, encapsulation efficiency, quercetin release and biological interactions with cardiac cells regarding nanoparticle association, and internalization and protective capability against relevant challenges). A better delivery of quercetin was achieved when encapsulated versus free. When the cells were challenged with antimycin A, it resulted in lower mitochondrial O_2_^−^ (4.65- vs. 5.69- fold) and H_2_O_2_ rate production (1.15- vs. 1.73- fold). Similarly, under hypoxia-reoxygenation injury, a better maintenance of cell viability was found (77 vs. 65%), as well as a reduction of thiol groups (~70 vs. 40%). Therefore, the delivery of encapsulated quercetin resulted in the preservation of mitochondrial function and ATP synthesis due to its improved oxidative stress suppression. The results point to the potential of this strategy for the treatment of oxidative stress-based cardiac diseases.

## 1. Introduction

Cardiac ischemia-reperfusion (I-R) injury is the paradoxical cellular damage upon reperfusion of ischemic tissue. This condition is to be considered a main contributor to heart damage in clinical conditions such as acute myocardial infarction or sudden death after a cardiac transplant [1, 2]. The pathophysiological mechanisms of I-R injury are complex and incompletely understood, and an effective treatment is currently unavailable [3]. It is relevant to note that mitochondrial reactive oxygen species (ROS) and calcium overload are considered key triggers of I-R damage.

Ischemia-related mitochondrial dysfunction is thought to result in substantial anion superoxide (O_2_^−^) production upon the reintroduction of oxygen. Both increased ROS and mitochondrial Ca^2+^ overload triggers the opening of the mitochondrial permeability transition pore (mPTP), leading to cell damage and a permanent loss of ATP production [4].

Under these circumstances, the prevention of increased ROS in cells may therefore impede the effects associated with an I-R injury. Options to reduce ROS production can be found in naturally occurring flavonoids, due to their antioxidant properties. Among the flavonoids, quercetin is one of the most abundant dietary flavonoids [5], having ROS scavenging capabilities and inhibiting ERK, MAP kinase, and Bmi-1 under ROS-generated cardiomyopathy [6]. The latter quercetin effects occur due to the activation of SIRT1, which is also related to the transcription of the antioxidant enzymes superoxide dismutase and catalase [7]. However, such effects have been found in human studies, with limited results [8]. This occurs in light of the low bioavailability and high metabolization rate of quercetin [9]. Therefore, protecting quercetin from degradation and increasing its bioavailability are desired qualities that can be achieved by its encapsulation in nanoparticles (NPs). Poly(lactic-co-glycolic) acid (PLGA) NPs are a performant platform, due to their biocompatibility, capability of tuning a controlled release rate for the encapsulated active compound, and high versatility for surface functionalization [10]. For example, this platform has been used to deliver cyclosporine A, an inhibitor of the opening of the mPTP, during cardiac damage, showing promising results [11].

The purpose of this study is to assess the delivery capability of quercetin from PLGA NPs into H9c2 cells, a surrogate model of cardiac cells, in a model of hypoxia-reoxygenation (H-R) injury. The results confirm a superior protection capability of PLGA-quercetin NP with respect to free quercetin. These results are based on improved antioxidant properties, decreasing cell death after H-R injury and preserving mitochondrial membrane potential (ΔΨ_*m*_) and ATP synthesis. The results are also being studied in light of the NP internalization dynamics and the quercetin release rate, highlighting their potential use as a therapeutic strategy against I-R injury.

## 2. Materials and Methods

### 2.1. Reagents

All chemical reagents, cell culture media and supplements, and fluorescent probes were purchased from Sigma-Aldrich (St. Louis, MO, USA), unless otherwise stated.

### 2.2. Nanoparticle Synthesis

PLGA nanoparticles (NPs) were synthetized through an oil-in-water protocol. Briefly, 40 mg of 50 : 50 poly(DL-lactide-co-glycolide) (PLGA, #B6010-2, LACTEL, Birmingham, LA, USA) were dissolved in 2 mL of acetone (J.T. Baker, CAS 67-64-1). Then, the solution was added dropwise into a flask with 8 mL of ultrapure H_2_O pH 3.5 under probe sonication (Qsonica Q700, 1/8^″^ tip) at 20% amplitude for 2 minutes in an ice bath. Afterwards, the NP solution was added to 30 mL of poly(vinyl alcohol) (PVA, #341584, CAS 9002-89-5) 0.1% (*w*/*v*) under magnetic stirring. Three hours later, the NPs were recovered via a centrifugation and washing procedure. PLGA-quercetin or PLGA-FITC NPs were obtained with the same protocol, mixing either quercetin (#Q4951, CAS 117-39-5) or fluorescein isothiocyanate (FITC, #F3651, CAS 27072-45-3), respectively, with PLGA when it is in the acetone.

### 2.3. Nanoparticle Characterization

#### 2.3.1. Particle Size Distribution

Transmission electron microscopy (TEM) was used to quantify the particle size distribution (PSD) of dry NPs. The PSD was constructed by measuring the Feret diameter of several individual NPs. Measurements were done in a FEI-TITAN 80-300 kV with a 300 kV electron beam. Samples were mounted on Cu grids coated with lacey carbon. Dynamic light scattering (DLS) was used to quantify the PSD of NPs in ultrapure H_2_O. Their PSD was obtained from the NP hydrodynamic diameter ensemble, which was determined by fitting the intensity autocorrelation function with the Contin method. Measurements were done in a Malvern Zetasizer Nano ZS90 (Malvern Instruments, Malvern, UK).

#### 2.3.2. Surface Charge

Electrophoretic light scattering (ELS) was used to determine the zeta potential of the NPs dispersed in ultrapure H_2_O using the Smoluchowski approximation. Measurements were done in a Malvern Zetasizer Nano ZS90 (Malvern Instruments, Malvern, UK).

#### 2.3.3. Quercetin Encapsulation Efficiency

Absorbance measurements were done to quantify the quercetin encapsulated in the PLGA-quercetin NPs, as well as quercetin left in the supernatant after the centrifugation and washing procedure. The NPs were dissolved with DMSO, and the supernatant was thoroughly vortexed previous to the measurement. Absorbance was assessed at 380 nm, and quercetin quantification was done with respect to a calibration curve. Measurements were done in a microplate fluorescence spectrophotometer Synergy HT (BioTek Instruments, Winooski, VT, USA). Quercetin encapsulation efficiency was calculated as

(1) $$\begin{array}{c} \text{EE}\left[\%\right]=100*\frac{\text{ mg of Querceti}{\text{n}}_{\text{encapsulated}}}{\text{mg of }{\text{Quercetin}}_{\text{encapsulated}}+{\text{Quercetin}}_{\text{supernatant}}}. \end{array}$$

#### 2.3.4. Quercetin Release from Nanoparticles

PLGA-quercetin NPs were suspended in 10 mL of Tyrode (in mM: 128 NaCl, 0.4 NaH_2_PO_4_, 5 glucose, 5.4 KCl, 0.5 MgCl-6H_2_O, and 25 HEPES) either at pH 7.4 or at pH 5.5. The suspension was fractionated in microtubes with 1.2 mL and shaken on a Thermomixer comfort (Eppendorf AG, Hamburg, Germany) at 700 rpm and 37°C. Microtubes were recovered at specific times, recovering the NPs by centrifugation, discarding the supernatant, dissolving the NP pellet with DMSO, and quantifying quercetin at 380 nm with respect to a calibration curve. Quercetin release was determined by comparing each time measurement with the 0 h measurement. Measurements were done in a microplate spectrophotometer Synergy HT (BioTek Instruments, Winooski, VT, USA). The release profiles were fitted to a cumulative Weibull distribution:

(2) $$\begin{array}{c} r_{Q}\left(t\right)=1-\exp\left[-{\left(\frac{t}{t_{\text{scale}}}\right)}^{a}\right], \end{array}$$

where *r*_*Q*_(*t*) is the released quercetin as a function of time, *a* is the shape parameter, and *t*_scale_ is the exponential scale parameter.

#### 2.3.5. Encapsulated and Free Quercetin Stability

PLGA-quercetin NPs or quercetin were suspended in 9 mL of Tyrode (in mM: 128 NaCl, 0.4 NaH_2_PO_4_, 5 glucose, 5.4 KCl, 0.5 MgCl-6H_2_O, and 25 HEPES, pH 7.4). The suspension was fractionated in 1.2 mL microtubes and shaken on a Thermomixer comfort (Eppendorf AG, Hamburg, Germany) at 700 rpm and 37°C. Microtubes were recovered at specific times, and NPs were recovered by centrifugation, and then, supernatant was discarded, dissolving the NP pellet with DMSO. Quercetin was evaluated from an aliquot of the microtube. Both encapsulated and free quercetin were quantified from 300 to 600 nm in a Synergy HT microplate spectrophotometer (BioTek Instruments, Winooski, VT, USA).

### 2.4. Nanobiointeractions and Viability under H-R Injury to Cardiac Cells

#### 2.4.1. Cell Culture

Neonatal rat ventricular myoblast H9c2 cell line (CRL-1446) was purchased from ATCC (Manassas, VA, USA). Cells were grown in Dulbecco's modified Eagle's medium (DMEM) (D7777) and supplemented with 10% fetal bovine serum (FBS) (Biowest, Riverside, MO, USA) and 1x penicillin-streptomycin (Gibco, Dún Laoghaire, Dublin, Ireland) in a humidified incubator at 37°C with 5% CO_2_ and 95% air.

#### 2.4.2. Nanoparticle Association and Internalization

Nanoparticle association to H9c2 cells was determined in dose dependence and time dependence using a BD FACSCanto II flow cytometer (BD Biosciences, Heidelberg, Germany). Cells were incubated with PLGA-FITC NPs or FITC at different doses and times. Afterwards, cells were trypsinized and resuspended in Tyrode solution (in mM: 5.4 KCl, 128 NaCl, 0.4 NaH_2_PO_4_, 0.5 MgCl_2_, 25 HEPES, 1.0 CaCl_2_, and 5 glucose). Cell association of nanoparticles was determined by analyzing the change in fluorescence (FITC). Cells were gated on single events and viable cells. Gating and fluorescence analysis were done using FlowJo (Treestar, Oregon, USA).

Cell internalization of NPs was determined by seeding H9c2 cells into glass coverslips. Cells were incubated with PLGA-FITC NPs for 24 h, followed by fixation and staining of cellular structures. Briefly, after PLGA-FITC incubation, cells were fixed using 4% paraformaldehyde in PBS buffer for 25 min. Then, cells were blocked with 2% bovine serum in PBS for 20 min. Afterwards, the actin filaments of the cytoskeleton were stained with Alexa Fluor 568-conjugated phalloidin (Life Technologies, Grand Island, NY), and the nuclei were stained with DRAQ5 (Thermo Fisher) for 20 min at room temperature. Finally, glass coverslips were mounted onto glass slides with VECTASHIELD mounting media (Vector Laboratories, Burlingame, CA). Fluorescence was assessed by confocal microscopy, using a Leica TCS SP5 confocal microscope equipped with a D-apochromatic 63X, 1.2 NA, oil objective (Leica Microsystems, Wetzlar, Germany). FITC was assessed using an Ar laser at 488 nm (excitation), and emission was acquired at 517 nm with a bandwidth of 22 nm. Phalloidin was assessed using a He-Ne laser at 543 nm (excitation), and emission was acquired at 590 nm with a bandwidth of 40 nm. DRAQ5 was assessed using a He-Ne laser at 633 nm (excitation), and emission was acquired at 733 nm with a bandwidth of 65 nm. For all samples, optical section images were acquired as a Z-stack with 1 *μ*m between the stacks, from the bottom to the top of the cells. All settings were maintained unaltered between images and from all cell conditions. Additionally, the background noise levels, brightness, and contrast adjustments were applied the same for all images.

Time-dependent experiments were fitted to a logistic function:

(3) $$\begin{array}{c} y\left(t\right)=A1+\frac{\left(A2-A1\right)}{\left(1+{\left(t/t_{0}\right)}^{p}\right)}, \end{array}$$

where *A*1 is the basal fluorescence, *A*2 is the maximum measured fluorescence, *t* is the time, *t*_0_ is the time when 50% fluorescence between *A*1 and *A*2 is achieved, and *p* is a constant modulating the steepness of the logistic function.

Dose-dependent experiments were fitted to an exponential model:

(4) $$\begin{array}{c} y\left(x\right)=A+B\left(1-\exp^{x/k}\right), \end{array}$$

where *A* is the basal fluorescence of the cells, *B* is a normalization constant to the exponential model, *x* is the applied dose, and *k* is the exponential scale parameter.

#### 2.4.3. Hypoxia-Reoxygenation Model

**Figure 1:**
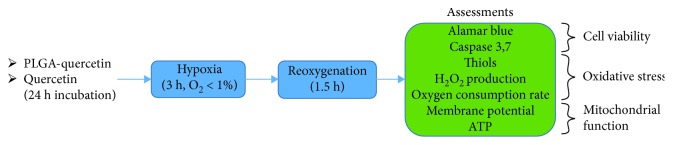
Schematic of the H-R protocol and assessments performed at a cellular and mitochondrial level.

A hypoxia challenge was given to trypsinized cardiomyoblasts, according to previous studies [12]. The cells were washed with Tyrode without glucose and incubated with an ischemic Tyrode (IT) solution simulating ischemic conditions (in mM: 135 NaCl, 8 KCl, 0.5 MgCl_2_, 0.33 NaH_2_PO_4_, 5 HEPES, 1.8 CaCl_2_, and 20 Na^+^ lactate, pH 6.8) [13] and transferred into an anaerobic chamber with an oxygen level < 1% at 37°C. After 3 h, cells were washed and incubated with Tyrode and 1.8 mM CaCl_2_ and transferred into an incubator in normoxic conditions (37°C with 5% CO_2_ and 95% air) for 1.5 h for reoxygenation, see Figure 1.

#### 2.4.4. Cell Viability and Cell Death Mechanisms

Cell viability was assessed under different doses of PLGA-quercetin or free quercetin. H9c2 cells were seeded at 1 × 10^3^ cells per well in 96-well plates and 72 h later were given treatments, incubated for 24 h. At the end of the incubation period, cell viability was determined by the Alamar blue viability test (Life Technologies, Carlsbad, CA). Measurements were done in a microplate fluorescence spectrophotometer Synergy HT (BioTek Instruments, Winooski, VT, USA). Dose-dependent viability was fitted to an exponential model:

(5) $$\begin{array}{c} y\left(x\right)=A-B\exp^{-x/k}, \end{array}$$

where *A* is the maximum viability of the cells after H-R injury, *B* is the viability amplitude, *x* is the applied dose, and *k* is the exponential scale parameter.

Apoptotic cells were measured by flow cytometry using Annexin V and 7-AAD staining. Briefly, cells were resuspended in Tyrode solution with 2.5 mM Ca^2+^, stained with an Annexin V Apoptosis Detection Set PE-Cy7 (eBioscience), and incubated for 10 min. Then, they were washed and resuspended in Tyrode solution with 2.5 mM Ca^2+^, stained with 7-AAD, and incubated for 5 min. Stained cells were then analyzed by flow cytometry using a FACSCanto II (BD Biosciences). Fluorescence compensation was performed postacquisition by using FlowJo V10.0 (Treestar). Apoptotic cells were identified as Annexin V (+)/7-AAD (-).

For caspase activity measurements, H9c2 cells were seeded at 1 × 10^3^ cells per well in 96-well plates and 72 h later were given treatments of PLGA-quercetin or free quercetin. After 24 h of incubation, the activities of caspases 3 and 7 were measured using the Caspase-Glo 3/7 assay (Promega, Madison, WI, USA), according to the manufacturer's protocols. Measurements were done in a microplate fluorescence spectrophotometer Synergy HT (BioTek Instruments, Winooski, VT, USA).

### 2.5. Oxidative Stress and Mitochondrial Function

#### 2.5.1. Reactive Oxygen Species Assessment

Mitochondrial anion superoxide (O_2_^−^) production was assessed in cardiomyoblasts with MitoSOX Red (Thermo Fisher Scientific). Briefly, cells were detached with Trypsin (L0931, Biowest, Missouri, USA); then, the cells were recovered by centrifugation and incubated in Tyrode with 5 *μ*M MitoSOX for 30 min at 37°C. Then, the cells were washed with Tyrode and 2.5 mM CaCl_2_ solution. Finally, each sample was stimulated with 20 *μ*g/mL antimycin A (AA) and measured 60 min later with a BD FACSCanto II flow cytometer (BD Biosciences, Heidelberg, Germany). Hydrogen peroxide was measured in cardiomyoblasts with Amplex Red (Thermo Fisher Scientific). Briefly, the cells were detached with Trypsin (L0931, Biowest, Missouri, USA); then, the cells were recovered by centrifugation and resuspended in a respiratory medium (in mM: 150 sucrose, 50 KCl, 2 KH_2_PO_4_, and 20 Tris-HCl, pH 7.3) with 40 *μ*M digitonin, 50 *μ*M Amplex Red, and 1.5 U/mL horseradish peroxidase. Measurements were done in a microplate fluorescence spectrophotometer Synergy HT (BioTek Instruments, Winooski, VT, USA). Data was fitted to a Gaussian model:

(6) $$\begin{array}{c} y\left(x\right)=y_{0}+Ae^{\left(-0.5{\left(\left(x-x_{c}\right)/w\right)}^{2}\right)}, \end{array}$$

where *y*_0_ is the offset, *A* is the amplitude, *x* is the dose of quercetin, *x*_*c*_ is the center of the peak, and *w* is the width of the peak proportional to its full width at half maximum (FWHM).

#### 2.5.2. Mitochondrial Respiration

Mitochondrial oxygen consumption rate (OCR) and membrane potential (ΔΨ_*m*_) were measured in parallel with an Oroboros Oxygraph-2k. For mitochondria measured from H9c2 cells, they were obtained after cell trypsinization, collection by centrifugation, and application of 40 *μ*M of digitonin. The experiments were carried out in a respiration assay medium containing 125 KCl, 10 HEPES-HCl, and 3 KH_2_PO_4_ (in mM) with pH 7.3. State 4 respiration was measured in the presence of 10 mM-2 *μ*g/mL succinate-rotenone. Maximal respiration was determined with 0.08 *μ*M of carbonyl cyanide-4-(trifluoromethoxy)phenylhydrazone (FCCP). The mitochondrial membrane potential was measured in parallel by fluorometry using 5 *μ*M safranine [14].

#### 2.5.3. Thiol Determination

Cells were detached with trypsin, washed with phosphate-buffered saline, and counted in the hemocytometer using the trypan blue exclusion dye. Cells (105) were suspended in Tris-HCl 200 mM pH 8.1, SDS 1%, EDTA 10 mM; after solubilization, 5,5′-dithiobis (2-nitrobenzoic acid) (DTNB) was added to reach 1 mM in the reaction (final volume 0.2 mL). After 10 minutes of color developing, absorbance was recorded at 412 nm using a multiwell plate reader. The extinction coefficient used is 14140 (that of the product of the reaction: 2-nitro-5-thiobenzoate anion at pH 8.1) to calculate the molar content of sulfhydryl groups. The molar determinations were corroborated using N-acetyl-L-cysteine in a calibration curve.

#### 2.5.4. ATP Assay

Intracellular ATP content was measured using the CellTiter-Glo Luminescent Assay (Promega, Madison, USA), according to the manufacturer's protocol. The ATP content is expressed as luminescence relative units (LRU). Measurements were done in a microplate fluorescence spectrophotometer Synergy HT (BioTek Instruments, Winooski, VT, USA).

#### 2.5.5. Statistical Analysis

All results were obtained from three independent (*n* = 3) measurements, unless otherwise stated. Data were analyzed by a one-way ANOVA followed by a Tukey multiple comparison tests. Data are presented as mean ± SEM (standard error of the mean). A *P* value < 0.05 was considered of statistical significance.

## 3. Results

### 3.1. Spherical PLGA-Quercetin NPs Encapsulate Quercetin Efficiently and Have a pH-Dependent Sustained Release

**Figure 2:**
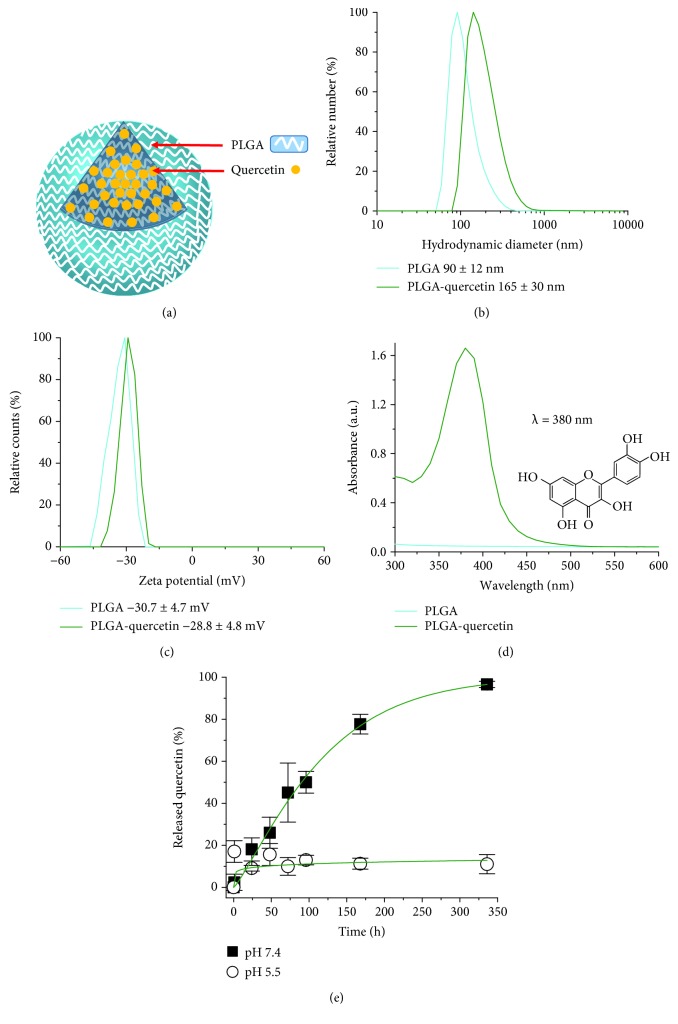
Physicochemical characterization of PLGA-quercetin NPs. (a) Cartoon of expected PLGA-quercetin structure. Representative plots of (b) PSD measured by DLS, (c) surface charge measured by ELS, and (d) encapsulation of quercetin in NPs by absorbance spectroscopy. (e) Release profile of quercetin from PLGA-quercetin NPs as a function of time and pH. Fittings (green lines) were based on equation (2).

PLGA NPs have a sphere-like morphology with an average Feret diameter of 90 ± 8.9 nm. Similarly, PLGA-quercetin NPs have a sphere-like morphology with an average Feret diameter of 157 ± 12.8 nm. See Supplementary Figure 1 for representative micrographs. A schematic of the NP is presented in Figure 2(a). When dispersed in water, PLGA and PLGA-quercetin NPs have an average hydrodynamic diameter of 90 ± 3 nm and 165 ± 7.5 nm, respectively, as evaluated from number distribution; see Figure 2(b) for a representative plot. The average surface charge of the PLGA and PLGA-quercetin NPs were −30.7 ± 1.2 and −28.8 ± 1.2 mV, respectively; see Figure 2(c) for a representative plot. By dispersion of PLGA-quercetin in DMEM culture media or DMEM+10% FBS, the cell culture media of these cells revealed a 17 and 3% increase in hydrodynamic diameter and a 21 and 9% increase in *ζ*-potential, respectively.  Table 1 summarizes all these data.

Quercetin (see molecular representation in the inset of Figure 2(d)) was incorporated into PLGA NPs with an encapsulation efficiency and drug loading of 98.15 ± 0.5% and 7.48 ± 1.87%, respectively, as evaluated at its absorption wavelength of 380 nm, using equation (1); see Figure 2(d) for a representative plot. The release of quercetin from PLGA NPs was studied in physiological media at pH 7.4 and 5.5 (see Figure 2(e)) showing a controlled release based on pH. At pH 7.4, the release was sustained and reached 100% at 336 h (2 weeks), while at pH 5.5, the maximum release was 10%, reaching this level in the first hour and staying as such afterwards. The release profiles were fitted to a Weibull distribution, where the *t*_scale_ at pH 7.4 is 122 h, and for pH 5.5, it is very high (2*e*9 h), which is interpreted as not having a reasonable biological release time.

### 3.2. Nanoparticle Association and Internalization Dynamics on Cardiac Cells

**Figure 3:**
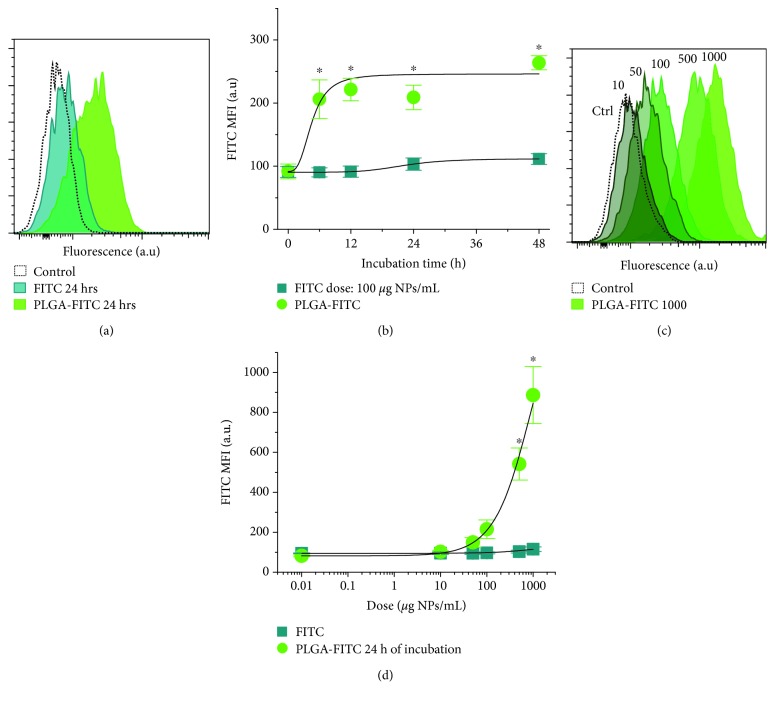
Association of PLGA-FITC NPs to H9c2 cells. (a) Representative FITC and PLGA-FITC when incubated with 100 *μ*g/mL for 24 h, (b) where time dependence shows an increase in fluorescence for PLGA-FITC after 6 h incubation; lines are fits to equation (3). (c) Representative dose dependence increase of fluorescence after 24 h incubation of PLGA-FITC, (d) which is not observed for FITC incubation; solid lines were fitted to equation (4). Statistical significance versus not treated (NT) cells, denoted as ^∗^, means *P* < 0.05. In (d), NT cells are denoted with 0.01.

The capability of PLGA NPs to associate with H9c2 cells was assessed by dose and time dependence. First, a dose of 100 *μ*g/mL of PLGA-FITC was selected to study the time-dependent association. At 24 h incubation, it was clearly observed that only NP incubation yielded a clear fluorescence increase (see Figure 3(a)), indicating NP association to the cell. This NP association into H9c2 cells saturated at around 6 h; see Figure 3(b). Fitting to equation (3) indicated a 50% NP association at 4.51 h and 22.15 h for FITC. Then, different doses of PLGA-FITC were incubated with the cells at 24 h; see Figure 3(c). A continuous dose-dependent NP-cell association was studied up to 1000 *μ*g/mL, where equimolar doses of FITC did not achieve a noticeable cell association with respect to NPs; see Figure 3(d). Fitting to equation (4) showed a dose scale of 769.23 *μ*g/mL for PLGA-FITC and 666.67 *μ*g/mL for FITC.

**Figure 4:**
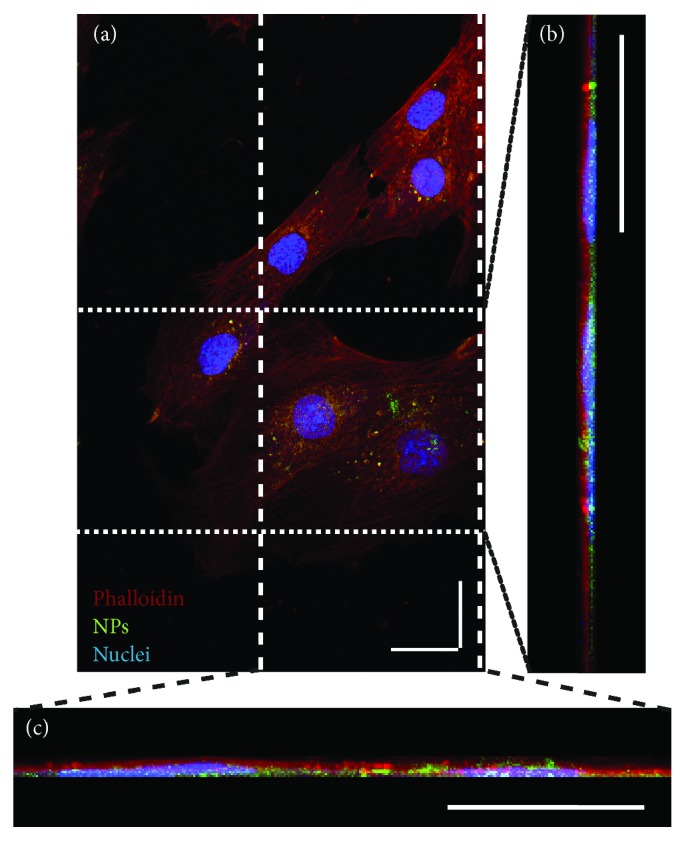
NP internalization into H9c2 cells. Representative fluorescence images, acquired by laser scanning confocal microscopy, of the incorporation of PLGA-FITC NP in H9C2 cells after 24 h of incubation. Staining of cells is as follows: blue: DRAQ5-stained nuclei; red: Alexa Fluor 568-conjugated phalloidin-stained actin filaments; and green: FITC-labeled PLGA NPs. (a) Representative *x*, *y* projection of an optical section of the cells. (b, c) Orthogonal projections *y*, *z* and *x*, *z*, reconstructed from the optical section stacks, acquired with 1 *μ*m of distance between each stack. Scale bars: 25 *μ*m.

The internalization of NPs was assessed by incubating H9c2 with PLGA-FITC. After 24 h of incubation, PLGA NPs encapsulating FITC are observed in cells; see Figure 4(a) for a representative *x*, *y* optical section. Regarding orthogonal projections in the planes *y*, *z* and *x*, *z*, Figures 4(b) and 4(c), respectively, demonstrate how NPs internalize the cells. FITC fluorescence was observed inside the cells only when it was encapsulated in NPs; see Supplementary Figure 2A-C. The average fluorescence per cell was quantified and found to increase at a dose-dependent rate for NPs; see Supplementary Figure 2D, where a fit to equation (4) showed a dose scale of 238.1 *μ*g/mL. Similar to the association data from flow cytometry, only NPs exerted a significant fluorescence increase, even at the highest FITC dose; see Supplementary Figure 2E.

### 3.3. Reduction of Mitochondrial ROS in H9c2 Cells with PLGA-Quercetin NPs

**Figure 5:**
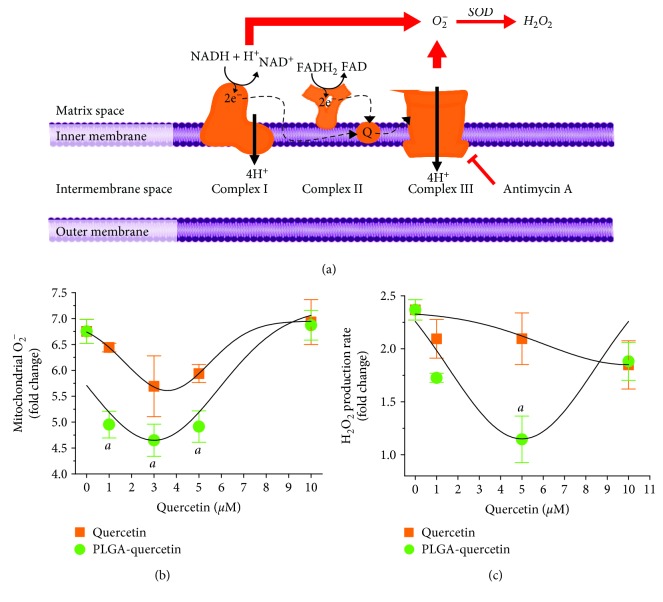
Assessment of mitochondrial O_2_^−^ and H_2_O_2_ production in H9c2 cells as a response to AA after 24 h quercetin or PLGA-quercetin treatments. (a) Description of the ROS production in the mitochondria due to AA stimulus. (b) Mitochondrial O_2_^−^ production. PLGA-quercetin shows a better dose-dependent O_2_^−^ suppression. (c) Mitochondrial H_2_O_2_ production rate. Treatments with quercetin or PLGA-quercetin are dose-dependent, with PLGA-quercetin presenting a better reduction of O_2_^−^ and H_2_O_2_. In (b) and (c), lines were fitted to equation (6). Statistical significance versus H-R cells, denoted as ^a^, means *P* < 0.05.

The cells were treated for 24 h with different doses of quercetin, either encapsulated or free. The cells were then subjected to a pharmacological ROS stimulus, using AA, which blocks the Complex III of the electron transport chain (ETC) of the mitochondria, thus increasing mitochondrial O_2_^−^ formation through Complexes I and III [15]. Mitochondrial O_2_^−^ is then transformed into H_2_O_2_ by superoxide dismutase (SOD); see Figure 5(a).

It is clearly observed that treatment with PLGA-quercetin is more effective for reducing the production of O_2_^−^ due to AA. The effect of AA is to increase the production of O_2_^−^ 6.75-fold, and its suppression is not related to the NP material, PLGA, as observed in Supplementary Figure 3A. Of the different treatments, the most effective dose for O_2_^−^ suppression was 3 *μ*M quercetin, free or encapsulated, reducing the ROS production down to 5.69-fold and 4.65-fold, respectively; see Figure 5(b). Treatments with PLGA-quercetin at 1, 3, and 5 *μ*M yielded a statistically significant reduced O_2_^−^ production at a rate of 4.95-, 4.65-, and 4.91-fold, respectively, all of which were lower than 3 *μ*M free quercetin. Equation (6) was used to fit both treatments, suggesting that the best dose to reduce O_2_^−^ production was 3.6 *μ*M. The production rate of H_2_O_2_ was assessed, showing that the AA stimulus could increase it 2.37-fold; see Figure 5(c). Treatment with quercetin in doses of 1-10 *μ*M showed a modest reduction down to 1.84-fold at 10 *μ*M, while treatments with encapsulated quercetin at 1 and 5 *μ*M reduced the production down to 1.73- and 1.15-fold, respectively. Indeed, the treatment with PLGA-quercetin at 5 *μ*M showed a statistically significant decrease with respect to untreated H9c2 cells stimulated with AA. Equation (6) points out that for PLGA-quercetin its best H_2_O_2_ suppression is at 5 *μ*M; the available data suggests that quercetin has its best suppression effect at 10 *μ*M. The reduction in the H_2_O_2_ production rate with encapsulated quercetin was not due to the NP material; see Supplementary Figure 3B. These differences in the efficiency of ROS quenching were assessed through the stability of encapsulated versus free quercetin incubated over 168 h. Encapsulated quercetin was stable, presenting a peak at 380 nm; see Supplementary Figures 4A and 4B. Free quercetin started to show an absorbance peak at 330 nm after 24 h of incubation, increasing its peak with time while diminishing it at 380 nm; see Supplementary Figures 4A and 4C.

### 3.4. Cardioprotection and Mitochondrial Function in Cardiac Cells Treated with PLGA-Quercetin NPs during H-R Injury

**Figure 6:**
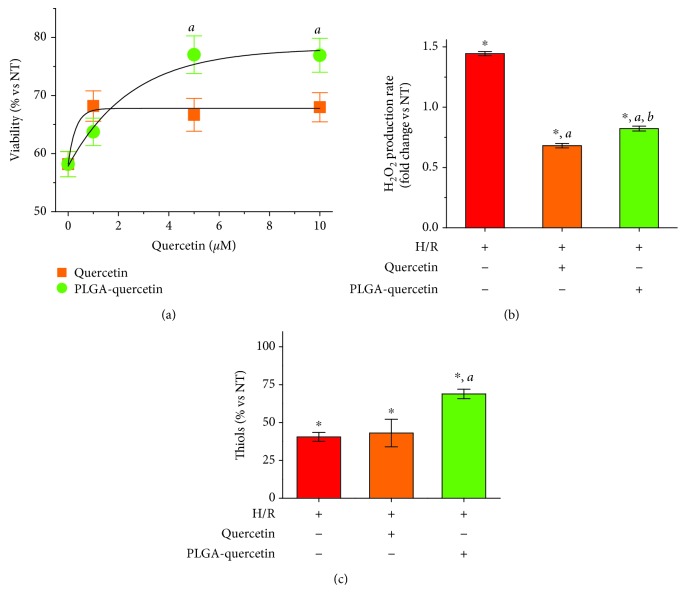
Protection of PLGA-quercetin against H-R in H9c2 cells. (a) Viability of H9c2 cells treated 24 h with quercetin or PLGA-quercetin treatments. Lines were fitted to equation (5). (b) H_2_O_2_ production rate. (c) Thiols. Note: for (b) and (c), the dose of quercetin, free or encapsulated, was 5 *μ*M. Statistical significance versus NT cells denoted as ^∗^, for H-R cells denoted as ^a^, and for quercetin-treated cells denoted by ^b^, means *P* < 0.05.

To further study the cardioprotective effects of PLGA-quercetin NPs under closer pathophysiological conditions, H9c2 cells were subjected to H-R after 24 h incubation with either encapsulated or free quercetin. Cells were subjected to hypoxia over 3 h, left into reoxygenation over 1.5 h, and finally analyzed for their viability; see Figure 1. The viability of the cells with incubated PLGA-quercetin with 5 and 10 *μ*M was rescued from 58 up to 77%, while treatment with quercetin only rescued cells up to around 67% regardless of the dose; see Figure 6(a). Fitting to equation (5) showed that the scale parameters were 0.3 and 2.51 for quercetin and PLGA-quercetin treatments, respectively. That is, treatment with PLGA-quercetin had a cell viability protection with an 8.37-fold higher dose of quercetin treatment. Incubation with PLGA NPs did not improve the viability of H-R cells; see Supplementary Figure 5. Observing that the best protected condition of cells with PLGA-quercetin is with a dose of 5 *μ*M quercetin, the remaining experiments were conducted at such dose, comparing only encapsulated versus free quercetin. No changes in apoptosis were observed, as assessed through Annexin V (see Supplementary Figures 6A and 6B) or activity of caspases 3 and 7 (see Supplementary Figure 6C). Necrosis was not a major factor in H-R. The production of H_2_O_2_ post-H-R was quenched by both quercetin treatments; see Figure 6(b). However, there was a clear preservation of thiols with the PLGA-quercetin treatment; see Figure 6(c).

**Figure 7:**
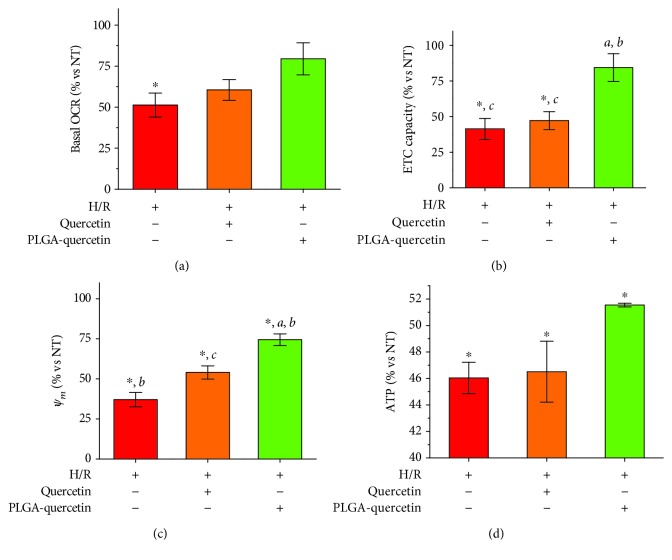
Protection of PLGA-quercetin against H-R in H9c2 cells: (a) basal OCR; (b) maximum ETC capacity; (c) mitochondrial membrane potential; (d) ATP production. All quercetin or PLGA-quercetin were done at 5 *μ*M. Statistical significance versus NT cells denoted as ^∗^, for H-R cells denoted as ^a^, for quercetin-treated cells denoted by ^b^, and for PLGA-quercetin-treated cells denoted by ^c^, means *P* < 0.05.

The potential protective effects of PLGA-quercetin were also explored on H9c2 cells after H-R. The OCR of cells treated with PLGA-quercetin yielded a better preservation; see Figures 7(a) and 7(b) for the basal OCR and maximum ETC capacity, respectively. Such preserved OCR values were also reflected in the improved ΔΨ_*m*_ (see Figure 7(c)) and ATP production; see Figure 7(d).

## 4. Discussion

The search for better treatments for cardiac dysfunction continues nowadays, even when several key mechanisms of the disease have been identified [16]. This is the case for diseases associated with an overproduction of ROS, for example, after an I-R event [3]. Solutions like the use of naturally occurring antioxidants, such as quercetin, are limited given their low bioavailability and high metabolic rate [9] and also due to their hydrophobic nature. In regard to these challenges, a potential alternative with a biocompatible NP, which can transport the antioxidant and deliver it efficiently into cardiac cells, has not been fully explored. In the design of a NP with a therapeutic *in vivo* aim, its size is of utmost importance, suggesting between 5 and 200 nm to avoid renal filtration or retention by the liver or spleen [17]. In the case of nanomedicine applications for cardiovascular diseases, there are plenty of NPs of around 200 nm that show positive results [16]. The results of this study show that PLGA-quercetin NPs have an average hydrodynamic diameter of 165 ± 7.5 nm, which is in the range of other reports of PLGA encapsulation of molecules with a similar molecular weight [18], and in excellent agreement with the diameter obtained from the micrographs of TEM showing spherical NPs. The surface charge for PLGA-quercetin NPs was −28.8 ± 1.08 mV, in line with the surface charge of PLGA NPs. In reality, however, NPs will interact with either amino acids or proteins during *in vitro* or *in vivo* scenarios. Such interactions will lead to the formation of a protein corona around the NPs, which in turn modifies their identity [19] through changes in size, surface charge, and the surface itself. These changes can modify the responses of cells, such as those of the immune system [20]. In this study, the incubation of PLGA-quercetin NPs to DMEM or its version supplemented with FBS used for cardiac cell culture resulted in small variations in terms of hydrodynamic diameter and *ζ*-potential in both cases, having the highest variations when incubated with the former. Such small variations, almost negligible when incubated with FBS-supplemented DMEM, could be a result of the low interaction sites of PLGA-quercetin. These results stand in contrast, for example, with studies of SiO_2_ NPs, smaller in size but of similar *ζ*-potential, which showed an increase in hydrodynamic diameter [21]. Therefore, these experiments seem to indicate, within the studied parameters, that the formation rate of the protein corona in PLGA-quercetin NPs is slower than for other particles of similar size and surface charge.

The release of quercetin from the NPs was studied in physiological media at a pH of 5.5 and 7.4, in order to model the release either at an endosome or in the cellular cytoplasm. The results indicate that at pH 5.5, the release of quercetin is limited to an average up to 10% regardless of time, indicating that this is a burst release. More importantly, this indicates that quercetin could avoid degradation when in an endosome through the encapsulation with PLGA. The latter is a desired trait during the period of time when the endosomal escape takes place [22]. The release dynamics at pH 7.4 suggest that quercetin delivery into the cytoplasm is sustained. No burst release of quercetin is observed at pH 7.4, as is verified by the 2.35% release after the first hour. Such limited release is desired at early time points to avoid drug side effects in secondary tissues [23] and allow the NP to arrive at the target tissue. The differences in the quercetin release from both conditions are reflected in the length of time (*t*_scale_) of the Weibull distribution, implying that for pH 5.5, the release will take an extremely long time, while for pH 7.4, a total of 63% will be released after 122 h. Molecule release parameters from NPs are conditioned to the molecule hydrophilic character and the NP material. For example, in a model of hollow PLGA NPs encapsulating siRNA complexes, the *t*_scale_ at pH 7.4 was extrapolated to 65.96 h [24], about half of that of the released quercetin in this study.

The association and internalization of PLGA to cardiac cells were studied in a dose- and time-dependent manner. In this study, the uptake, NP association to cellular membrane, and internalization of PLGA-FITC NPs in dose dependence showed an exponential growth tendency with a timescale of 769.23 *μ*g/mL, while the time dependence for the dose of 100 *μ*g/mL was saturated since 6 h with an estimated 4.51 h to reach half of the maximum uptake. Uptake dynamics are governed by the interactions of the NP system and the cell. In a study of SiO_2_, nano- and microparticle uptake into adult rat cardiomyocytes was quantified by Particle-Induced X-ray Emission (PIXE), a chemical element quantification technique recently used in nanosafety studies [25], and it was found that half maximum uptake was achieved in less than 2 h [21]. The SiO_2_ NPs had an average hydrodynamic diameter and surface charge similar to the NPs used in this study, and given that the same associative behavior was observed in the microparticles, this uptake difference can be attributed to the higher volume and surface area of adult rat cardiomyocytes, allowing for a faster uptake dynamic. This is influenced by the type of cell studied; for example, in A549 lung epithelial carcinoma cells, 40 nm fluorescent carboxylated polystyrene NPs reached half maximum uptake at 2 h within a 6 h study [26]. This observed faster kinetic with respect to this study can also be associated with the smaller NP size, with respect to that of this study, allowing for a faster cell uptake [17], as well as with the inherent faster uptake kinetic of cancerous cells. The internalization of PLGA-FITC NPs was assessed with a time scale of 238.1 *μ*g/mL as evaluated under confocal microscopy after 24 h. Therefore, according to a first approximation, out of the 769.23 *μ*g/mL uptaken NPs in H9c2 cells after 24 h of incubation, 30.95% were located around the middle of the cells. More precise information would be required to deduce the actual internalized quantity of NPs, such as the association dynamics. Nevertheless, this result implies that a fair number of NPs reach the central parts of these cells.

Quercetin, a potent antioxidant, can reduce ROS by means of its scavenger activity and can increase the activity of SIRT1 [7]. This effect has been previously demonstrated in H9c2 cells administered with H_2_O_2_, with an optimal dose of 10 *μ*M [27]. In this study, the effect of PLGA-quercetin on ROS production was assessed in its capability to inhibit ROS that originated from the mitochondria, hence the use of AA to inhibit Complex III of the ETC and generate acutely ROS. With respect to mitochondrial O_2_^−^ production, both PLGA-quercetin and quercetin showed a bell-shaped inhibition centered around 3 and 3.6 *μ*M, respectively. This bell-shaped inhibition is consistent with the prooxidant capability of exogenous antioxidants, such as quercetin, when in excess [28]. The assessed high doses in this study correlate with mitochondrial dysfunction [29]. In this study, prooxidant effects were observed in both O_2_^−^ and H_2_O_2_ productions at 10 *μ*M quercetin or PLGA-quercetin, except for quercetin in H_2_O_2_ production, where it seemed that the bell-shaped distribution had not yet reached its peak. The greater efficiency of PLGA-quercetin at inhibiting an overproduction of mitochondrial O_2_^−^ and H_2_O_2_ compared to its free counterpart in an *in vitro* setting, ~22 and 83% at 5 *μ*M, respectively, is due in part to the protection of encapsulated quercetin from degradation, which is likely an oxidation process [30, 31]. In order to reach the cytoplasm of the cell, the transport of NPs in a classical view includes the endocytosis by the cell and then its eventual escape from the endosome or lysosome [17]. However, there is recent evidence that negatively charged polystyrene or TiO_2_ NPs can enter cardiac cells through the formation of transient nanopores on the cell membrane [32, 33]. Given that PLGA-quercetin NPs have a negative surface charge, it is possible that this NP may enter cardiac cells through the formation of these reported nanopores. Additionally, according to the *in vitro* release profile of PLGA-quercetin, only 16.5% of quercetin is released at 24 h. A first approximation of the effective dose delivered into cardiac cells, assuming that 100% of the PLGA-quercetin NPs enter the cells and that only 16.5% of quercetin is released after 24 h of incubation, is that at this time there is only 16.5% available of the applied dose. Then, O_2_^−^ inhibition stayed at a similar level between effective doses of 0.165-0.825 *μ*M (applied doses of 1-5 *μ*M). A possible explanation for this similar level of O_2_^−^ inhibition, in other words a lack of further inhibition decrease within a 5-fold dose increase, is that there seems to be a limit to the quercetin delivery or quercetin availability to the mitochondria with this NP. Current clinical trials aiming to improve the outcome of patients with acute myocardial infarction have found limited results [34, 35]. Given this, the improved protection found in this study could become a key component in the search for efficient therapies that can deal with the excess ROS produced due to I-R injury. In the case of H_2_O_2_, which was measured in whole cells, there is a dose-dependent inhibition of up to 5 *μ*M of the applied dose, consistent with an increasing quercetin availability within the cytoplasm. A study of cerebral I-R in Wistar rats treated with PLGA-quercetin NPs showed a reduced mitochondrial-based ROS production in agreement with the present results [36]. In that study, the NPs were added to triphenyl phosphonium for NP targeting toward the mitochondria once inside the cell, showing a slight improvement in protection with respect to the overall results. These improvements could also be related to damaged tissue, where inflammation plays a role in increasing the accumulation of NPs [16, 37]. It will therefore be necessary to quantify the NP uptake to the damaged tissues, which will define the effective delivered dose *in vivo* in conjunction to its release rate.

The cells treated with free quercetin in the range of 1-10 *μ*M showed the same viability after H-R, ~67.5%, regardless of the dose, a 10% higher viability with respect to NT cells. Treatment with PLGA-quercetin at 5 and 10 *μ*M showed the same viability, 77%, or a 20% higher viability with respect to NT cells. Given that improved viability could be related to a reduction of apoptosis, the activity of executioner caspases 3 and 7 was assessed, showing similar activities for both free and nanoencapsulated quercetin. Therefore, the observed reduction of cytotoxicity by the NP was due to a reduction in cell necrosis. More thiols were preserved within the cells treated with PLGA-quercetin, pointing to a preservation of the mitochondrial ETC. This result, in addition to the lower H_2_O_2_ production rate, points to a lower oxidative stress, avoiding the opening of the mPTP due to the prevention of the binding of adenine nucleotide to the adenine nucleotide translocase, and therefore avoiding the release of apoptotic-cell-death-signaling factors such as cytochrome c [38]. Indeed, mitochondrial OCR and ΔΨ_*m*_ were better preserved in the PLGA-quercetin treatment. Aerobic ATP production in cells after H-R is expected to decrease as a function of mitochondrial dysfunction [13], as was found in this study, where only the treatment with PLGA-quercetin rescued 11.9% of the ATP production with respect to NT cells. Therefore, the improved cell viability rescued with PLGA-quercetin treatments is related with a better preservation of mitochondrial functions. In a recent study using siRNA to silence the mitochondrial calcium uniporter (MCU), the mitochondrial unit responsible for Ca^2+^ uptake, cardiac cells under the same H-R injury, showed 30% more viability and reduced activity for caspases 3 and 7 on the silenced cells [12]. This improved viability and reduced caspase activities could be mechanistically explained by the fact that the excess Ca^2+^ uptake rate in the mitochondria is responsible for increased ROS production. In other words, the reduced Ca^2+^ uptake by MCU silencing could have been enough to keep mitochondrial ROS production at a threshold that induced less mitochondrial dysfunction compared to the direct mitochondrial ROS inhibition in this study, hence reducing the ROS-induced ROS release [39], avoiding the activation of the apoptosis-signaling cascade. Nevertheless, as a strategy to reduce cytotoxic effects in an H-R injury to H9c2 cells, ROS inhibition has shown results in agreement with strategies aiming for MCU silencing and MCU inhibition *ex vivo* [12, 40]. Finally, the *in vivo* evaluation of the strategy presented here will assess its translational potential. Overall, this study suggests that the improved cell viability under H-R damage when treated with PLGA-quercetin is due to the improved delivery of the antioxidant, which prevents thiol oxidation in the cell, resulting in a preserved mitochondrial function, as observed by its OCR, ΔΨ_*m*_, and ATP production.

## 5. Conclusion

The potential of encapsulated quercetin, a PLGA-quercetin NP, was assessed in cardiac cells for the first time. In a pharmacological model of acute ROS production, induced by AA, PLGA-quercetin showed a potent ROS inhibition with respect to free quercetin. In an *in vitro* physiological model of H-R injury resembling an ischemia reperfusion event, it was found that PLGA-quercetin offers a better cell rescue, mainly due to lower oxidized thiols, maintaining the mitochondrial OCR and ΔΨ_*m*_, which sustain superior ATP production. Such results demonstrate the potential of such a strategy toward improved therapies for ROS-based cardiac diseases.

## Figures and Tables

**Table 1:** Physicochemical parameters of PLGA-quercetin NPs.

Parameter	PLGA	PLGA-quercetin	
Average Feret diameter (nm)	90 ± 8.8	156.7 ± 12.8	
Average hydrodynamic diameter (nm)	90 ± 3	165 ± 7.5 (ultrapure H_2_O), + 17%(DMEM incubation), + 3%(DMEM + 10%FBS incubation)	
Average zeta potential (mV)	−30.7 ± 1.2	−28.8 ± 1.08 (ultrapure H_2_O), − 21%(DMEM incubation), + 9%(DMEM + 10%FBS incubation)	
Quercetin encapsulation efficiency (%)	n/a	98.2 ± 0.42	
Release profile		pH: 7.4	5.5
50% quercetin release (h)	n/a	90 ± 17	>340
*t*_scale_ (h)	n/a	122 ± 23	2*e*9 ± 5*e*8

In average Feret diameter, *n* > 80. In average hydrodynamic diameter and average zeta potential, *n* up to 16. In release profile, dispersion was calculated from the error of fitting parameters. n/a: not applicable.

## Data Availability

The data used to support the findings of this study are available from the corresponding author upon request.

## Supplementary Materials

Supplementary Materials Supplementary Figure 1: representative TEM micrographs of (A) PLGA NPs and (B) PLGA-quercetin NPs. Scale bars of 100 nm. Insets: (A) representative TEM micrograph of PLGA NPs, scale bar of 200 nm; (B) representative TEM micrograph of PLGA-quercetin NPs, scale bar of 100 nm. Supplementary Figure 2: NP internalization into H9c2 cells after 24 h incubation. (A–C) Representative images of NT, FITC, and PLGA-FITC showing NPs can internalize into the cells. Merged images are composed of cell actin filaments (red), NP agglomerates (green), and nuclei (blue). Dotted yellow ellipses denote areas with NP accumulation. Scale bar of 40 *μ*m. (D) NPs internalize as a function of the dose, and lines were fitted to equation (4). Statistical significance versus untreated (NT) cells, denoted as ^∗^, means *P* < 0.05. In (D), NT cells are denoted with 1*e*-3. (E) FITC fluorescence, at equimolar incubation doses, is higher when encapsulated in PLGA NPs. Supplementary Figure 3: ROS quenching of PLGA NPs in H9c2 cells due to antimycin A (AA): (A) mitochondrial O_2_^−^ and (B) H_2_O_2_. Supplementary Figure 4: encapsulated and free quercetin stability incubated in physiological solution, pH 7.4. (A) 380 nm/330 nm absorbance ratio as a function of time. Data were normalized against control groups at 0 h. ^∗^Statistical significance between each groups at different times vs. its control at 0 h. ^#^Statistical significance between quercetin and PLGA-quercetin groups at specific times. (B) Selected absorbance spectra of encapsulated quercetin at 0, 48, and 168 h. (C) Selected absorbance spectra of free quercetin at 0, 48, and 168 h. Supplementary Figure 5: viability of H9c2 cells after 24 h of PLGA NP treatment, followed by the H/R. Supplementary Figure 6: apoptosis and necrosis of H9c2 cells after 24 h treatments, followed by H/R. (A) Representative dot plots of apoptosis and necrosis. (B) Apoptosis assessed by Annexin V. (C) Caspase 3/7 activity.

## References

[1] Garcia-Rivas G. J., Torre-Amione G. (2009). Abnormal mitochondrial function during ischemia reperfusion provides targets for pharmacological therapy. *Methodist DeBakey Cardiovascular Journal*.

[2] Kohlhauer M., Dawkins S., Costa A. S. H. (2018). Metabolomic profiling in acute ST-segment–elevation myocardial infarction identifies succinate as an early marker of human ischemia–reperfusion injury. *Journal of the American Heart Association*.

[3] Ruiz-Meana M., García-Dorado D. (2009). Pathophysiology of ischemia-reperfusion injury: new therapeutic options for acute myocardial infarction. *Revista Española de Cardiología*.

[4] Bernardi P., Vassanelli S., Veronese P., Colonna R., Szabó I., Zoratti M. (1992). Modulation of the mitochondrial permeability transition pore. Effect of protons and divalent cations. *Journal of Biological Chemistry*.

[5] Anand David A. V., Arulmoli R., Parasuraman S. (2016). Overviews of biological importance of quercetin: a bioactive flavonoid. *Pharmacognosy Reviews*.

[6] Dong Q., Chen L., Lu Q. (2014). Quercetin attenuates doxorubicin cardiotoxicity by modulating Bmi-1 expression. *British Journal of Pharmacology*.

[7] Hung C. H., Chan S. H., Chu P. M., Tsai K. L. (2015). Quercetin is a potent anti-atherosclerotic compound by activation of SIRT1 signaling under oxLDL stimulation. *Molecular Nutrition & Food Research*.

[8] Askari G., Ghiasvand R., Feizi A., Ghanadian S. M., Karimian J. (2012). The effect of quercetin supplementation on selected markers of inflammation and oxidative stress. *Journal of Research in Medical Sciences*.

[9] Formica J. V., Regelson W. (1995). Review of the biology of quercetin and related bioflavonoids. *Food and Chemical Toxicology*.

[10] Danhier F., Ansorena E., Silva J. M., Coco R., le Breton A., Préat V. (2012). PLGA-based nanoparticles: an overview of biomedical applications. *Journal of Controlled Release*.

[11] Ikeda G., Matoba T., Nakano Y. (2016). Nanoparticle-mediated targeting of cyclosporine A enhances cardioprotection against ischemia-reperfusion injury through inhibition of mitochondrial permeability transition pore opening. *Scientific Reports*.

[12] Oropeza-Almazán Y., Vázquez-Garza E., Chapoy-Villanueva H., Torre-Amione G., García-Rivas G. (2017). Small interfering RNA targeting mitochondrial calcium uniporter improves cardiomyocyte cell viability in hypoxia/reoxygenation injury by reducing calcium overload. *Oxidative Medicine and Cellular Longevity*.

[13] Zhao M., Sun L., Yu X. J. (2013). Acetylcholine mediates AMPK-dependent autophagic cytoprotection in H9c2 cells during hypoxia/reoxygenation injury. *Cellular Physiology and Biochemistry*.

[14] de Jesús García-Rivas G., Guerrero-Hernández A., Guerrero-Serna G., Rodríguez-Zavala J. S., Zazueta C. (2005). Inhibition of the mitochondrial calcium uniporter by the oxo-bridged dinuclear ruthenium amine complex (Ru_360_) prevents from irreversible injury in postischemic rat heart. *The FEBS Journal*.

[15] Drose S., Brandt U. (2008). The mechanism of mitochondrial superoxide production by the cytochrome *bc*
_1_ complex. *Journal of Biological Chemistry*.

[16] Lozano O., Torres-Quintanilla A., García-Rivas G. (2018). Nanomedicine for the cardiac myocyte: where are we?. *Journal of Controlled Release*.

[17] Blanco E., Shen H., Ferrari M. (2015). Principles of nanoparticle design for overcoming biological barriers to drug delivery. *Nature Biotechnology*.

[18] Ruiz-Esparza G. U., Wu S., Segura-Ibarra V. (2014). Polymer nanoparticles encased in a cyclodextrin complex shell for potential site- and sequence-specific drug release. *Advanced Functional Materials*.

[19] Lynch I., Salvati A., Dawson K. A. (2009). Protein-nanoparticle interactions: what does the cell see?. *Nature Nanotechnology*.

[20] Escamilla-Rivera V., Uribe-Ramírez M., González-Pozos S., Lozano O., Lucas S., de Vizcaya-Ruiz A. (2016). Protein corona acts as a protective shield against Fe_3_O_4_-PEG inflammation and ROS-induced toxicity in human macrophages. *Toxicology Letters*.

[21] Guerrero-Beltrán C. E., Bernal-Ramírez J., Lozano O. (2017). Silica nanoparticles induce cardiotoxicity interfering with energetic status and Ca^2+^ handling in adult rat cardiomyocytes. *American Journal of Physiology-Heart and Circulatory Physiology*.

[22] Martens T. F., Remaut K., Demeester J., de Smedt S. C., Braeckmans K. (2014). Intracellular delivery of nanomaterials: how to catch endosomal escape in the act. *Nano Today*.

[23] Sun Q., Radosz M., Shen Y. (2012). Challenges in design of translational nanocarriers. *Journal of Controlled Release*.

[24] Stigliano C., Aryal S., de Tullio M. D. (2013). siRNA-chitosan complexes in poly(lactic-co-glycolic acid) nanoparticles for the silencing of aquaporin-1 in cancer cells. *Molecular Pharmaceutics*.

[25] Lozano O., Mejia J., Masereel B., Toussaint O., Lison D., Lucas S. (2012). Development of a PIXE analysis method for the determination of the biopersistence of SiC and TiC nanoparticles in rat lungs. *Nanotoxicology*.

[26] Salvati A., Nelissen I., Haase A. (2018). Quantitative measurement of nanoparticle uptake by flow cytometry illustrated by an interlaboratory comparison of the uptake of labelled polystyrene nanoparticles. *NanoImpact*.

[27] Angeloni C., Spencer J. P. E., Leoncini E., Biagi P. L., Hrelia S. (2007). Role of quercetin and its in vivo metabolites in protecting H9c2 cells against oxidative stress. *Biochimie*.

[28] Bouayed J., Bohn T. (2010). Exogenous antioxidants—double-edged swords in cellular redox state: health beneficial effects at physiologic doses versus deleterious effects at high doses. *Oxidative Medicine and Cellular Longevity*.

[29] Ortega R., Garcia N. (2009). The flavonoid quercetin induces changes in mitochondrial permeability by inhibiting adenine nucleotide translocase. *Journal of Bioenergetics and Biomembranes*.

[30] Dall’Acqua S., Miolo G., Innocenti G., Caffieri S. (2012). The photodegradation of quercetin: relation to oxidation. *Molecules*.

[31] Mocek M., Richardson P. J. (1972). Kinetics and mechanism of quercetin oxidation. *Journal of the Institute of Brewing*.

[32] Miragoli M., Novak P., Ruenraroengsak P. (2013). Functional interaction between charged nanoparticles and cardiac tissue: a new paradigm for cardiac arrhythmia?. *Nanomedicine*.

[33] Savi M., Rossi S., Bocchi L. (2014). Titanium dioxide nanoparticles promote arrhythmias via a direct interaction with rat cardiac tissue. *Particle and Fibre Toxicology*.

[34] Cung T. T., Morel O., Cayla G. (2015). Cyclosporine before PCI in patients with acute myocardial infarction. *The New England Journal of Medicine*.

[35] Jaxa-Chamiec T., Bednarz B., Drozdowska D. (2005). Antioxidant effects of combined vitamins C and E in acute myocardial infarction. The randomized, double-blind, placebo controlled, multicenter pilot Myocardial Infarction and VITamins (MIVIT) trial. *Kardiologia Polska*.

[36] Ghosh S., Sarkar S., Choudhury S. T., Ghosh T., Das N. (2017). Triphenyl phosphonium coated nano-quercetin for oral delivery: neuroprotective effects in attenuating age related global moderate cerebral ischemia reperfusion injury in rats. *Nanomedicine: Nanotechnology, Biology and Medicine*.

[37] Ruiz-Esparza G. U., Segura-Ibarra V., Cordero-Reyes A. M. (2016). A specifically designed nanoconstruct associates, internalizes, traffics in cardiovascular cells, and accumulates in failing myocardium: a new strategy for heart failure diagnostics and therapeutics. *European Journal of Heart Failure*.

[38] Halestrap A. P., Pasdois P. (2009). The role of the mitochondrial permeability transition pore in heart disease. *Biochimica et Biophysica Acta (BBA) - Bioenergetics*.

[39] Zorov D. B., Juhaszova M., Sollott S. J. (2014). Mitochondrial reactive oxygen species (ROS) and ROS-induced ROS release. *Physiological Reviews*.

[40] García-Rivas G. J., Carvajal K., Correa F., Zazueta C. (2006). Ru_360_, a specific mitochondrial calcium uptake inhibitor, improves cardiac post-ischaemic functional recovery in rats in vivo. *British Journal of Pharmacology*.

