# Supplementary material for: Nanoencapsulated Quercetin Improves Cardioprotection during Hypoxia-Reoxygenation Injury through Preservation of Mitochondrial Function
Source: Oxid Med Cell Longev. 2019 Jun 24;2019:7683051. doi: 10.1155/2019/7683051 (PMC6612997; doi:10.1155/2019/7683051)

Supplementary Figure 1

A)

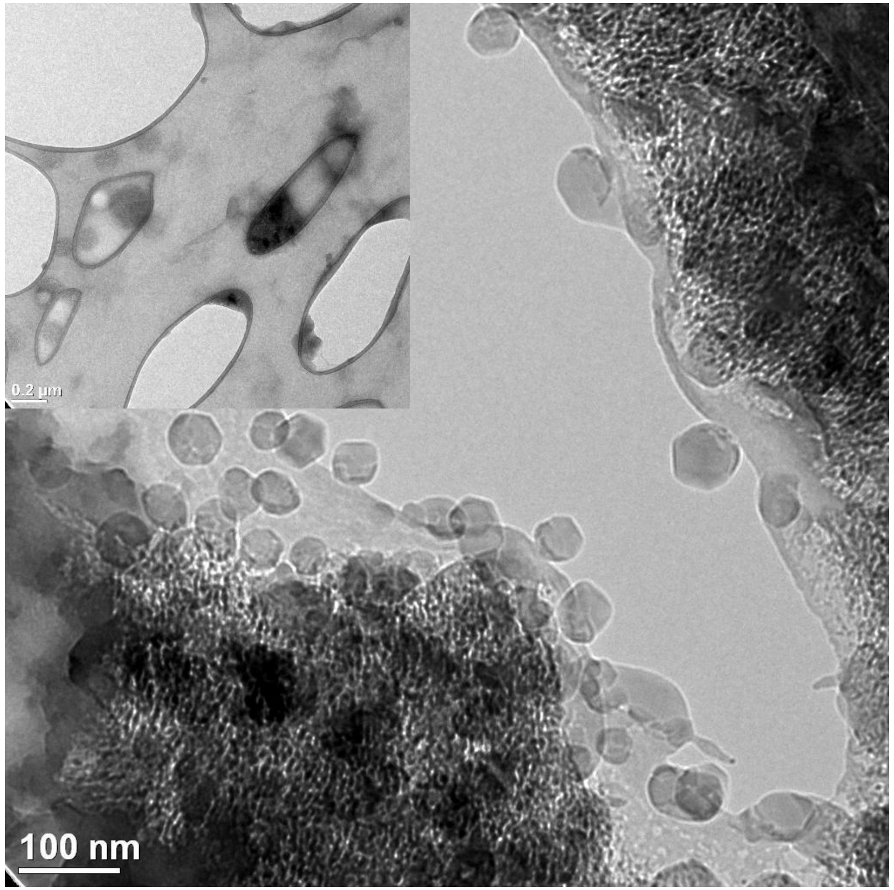

B)

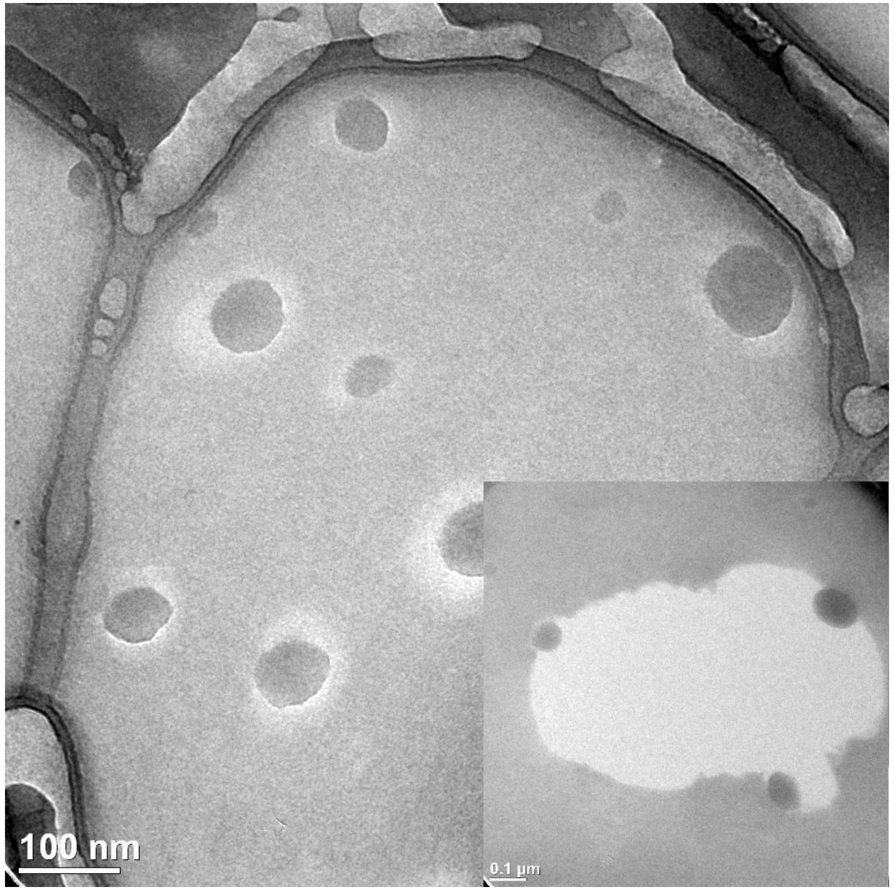

Supplementary Figure 2

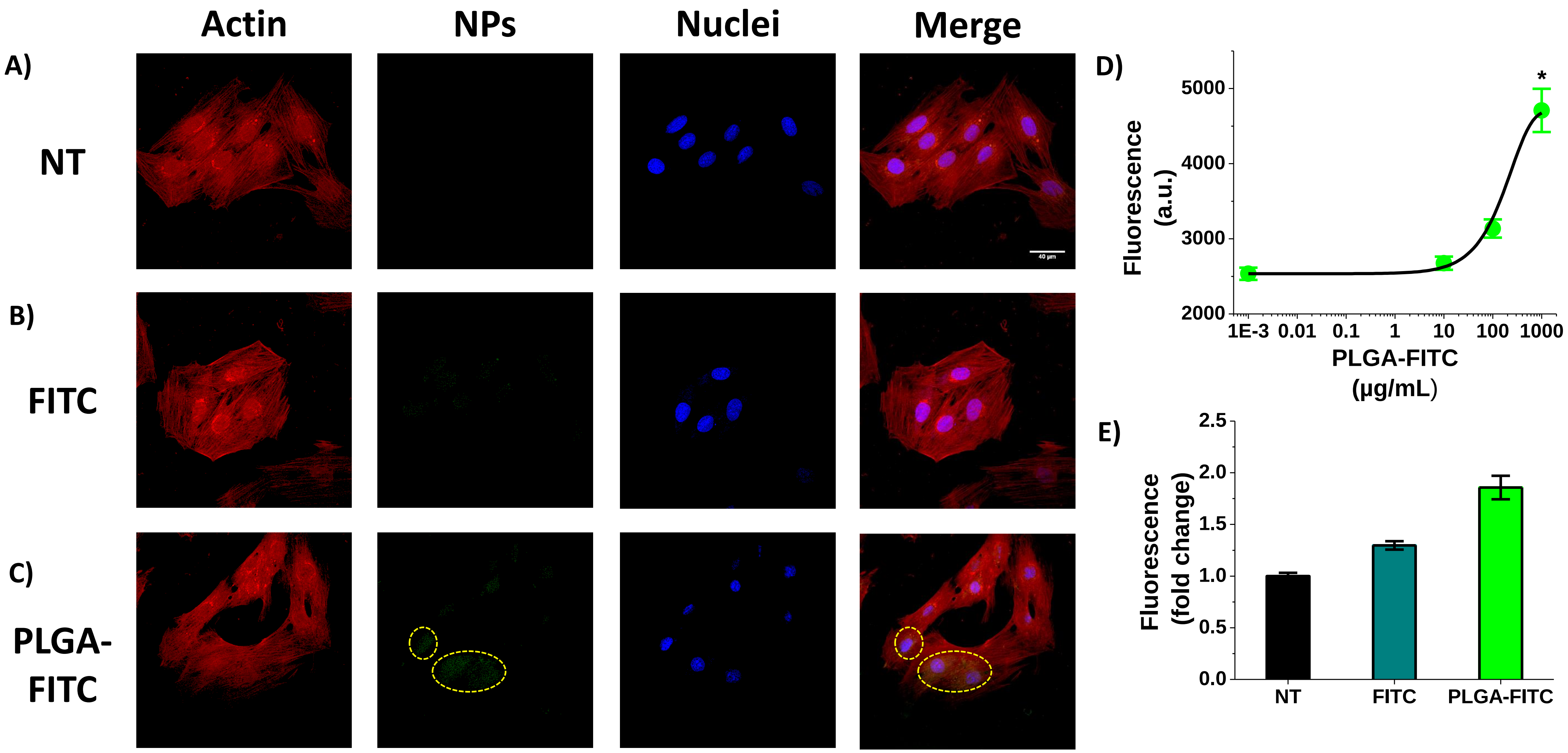

Supplementary Figure 3

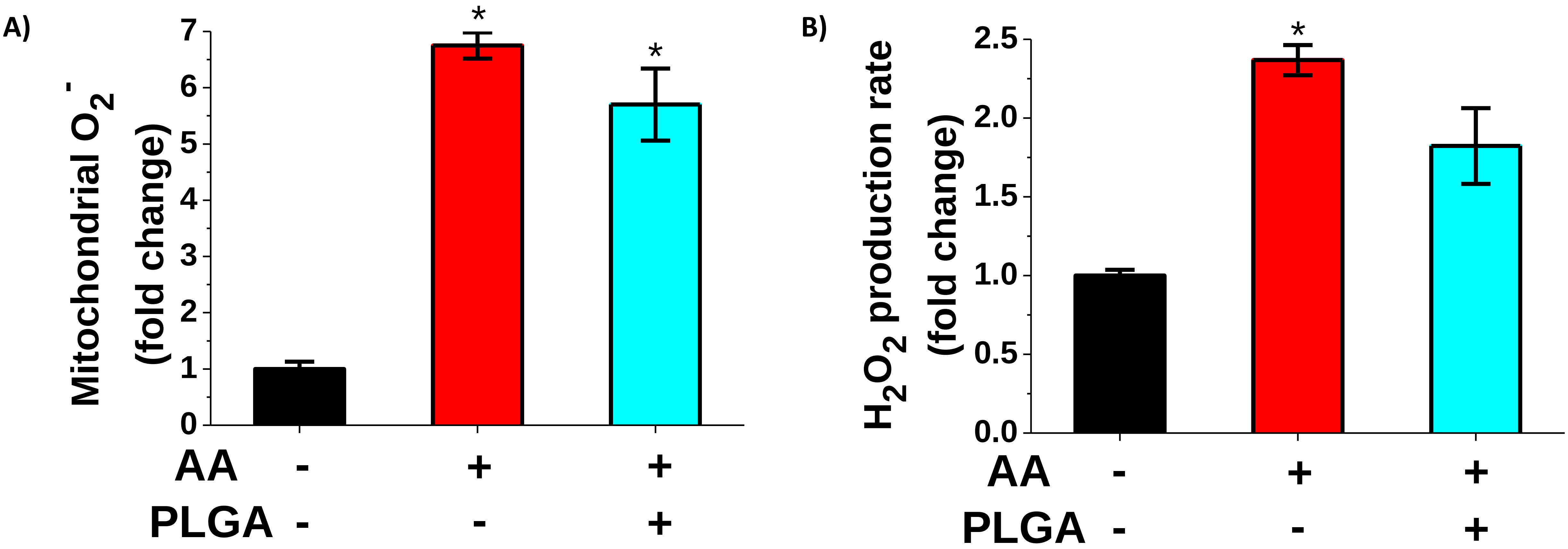

Supplementary Figure 4

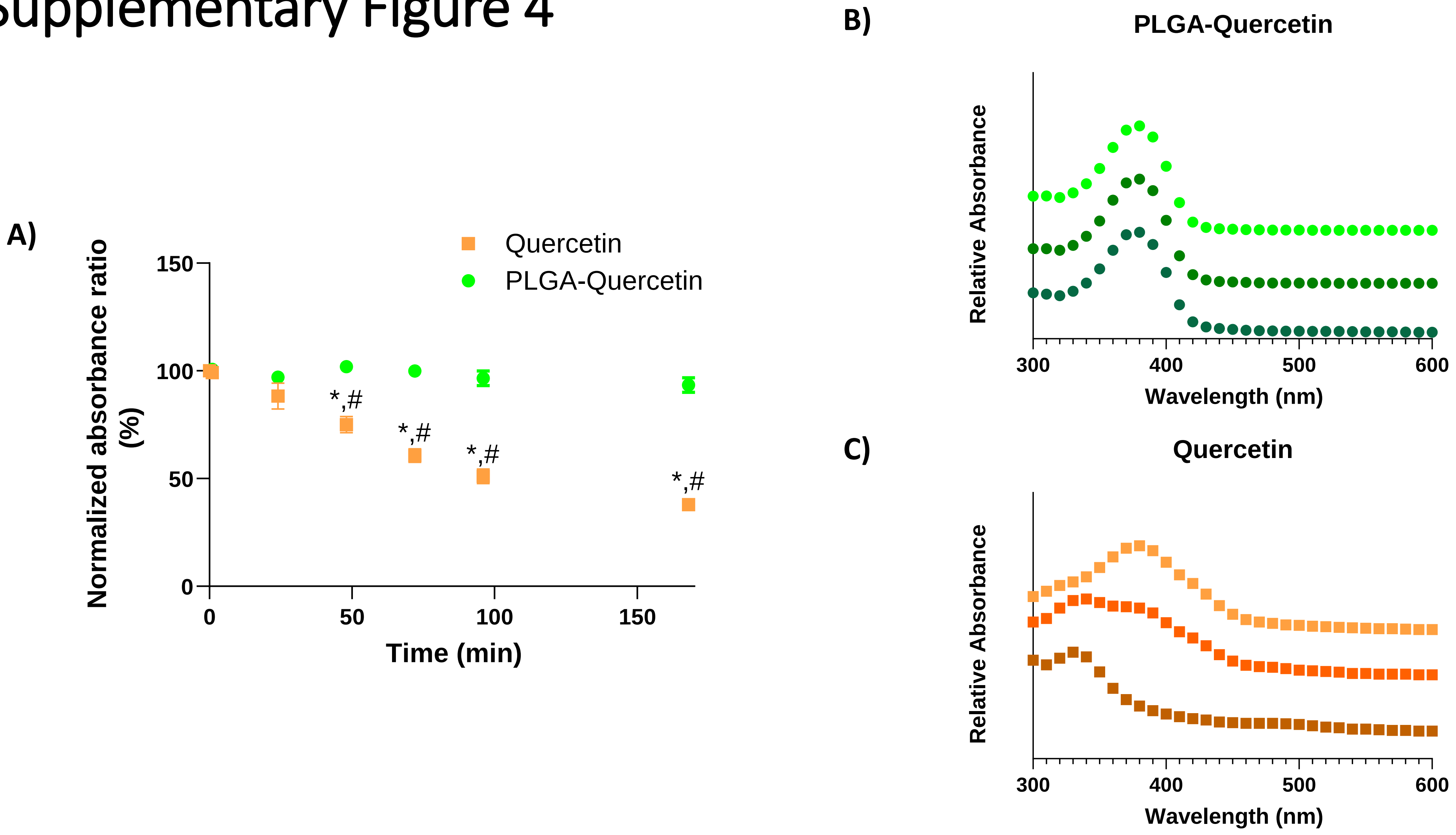

Supplementary Figure 5

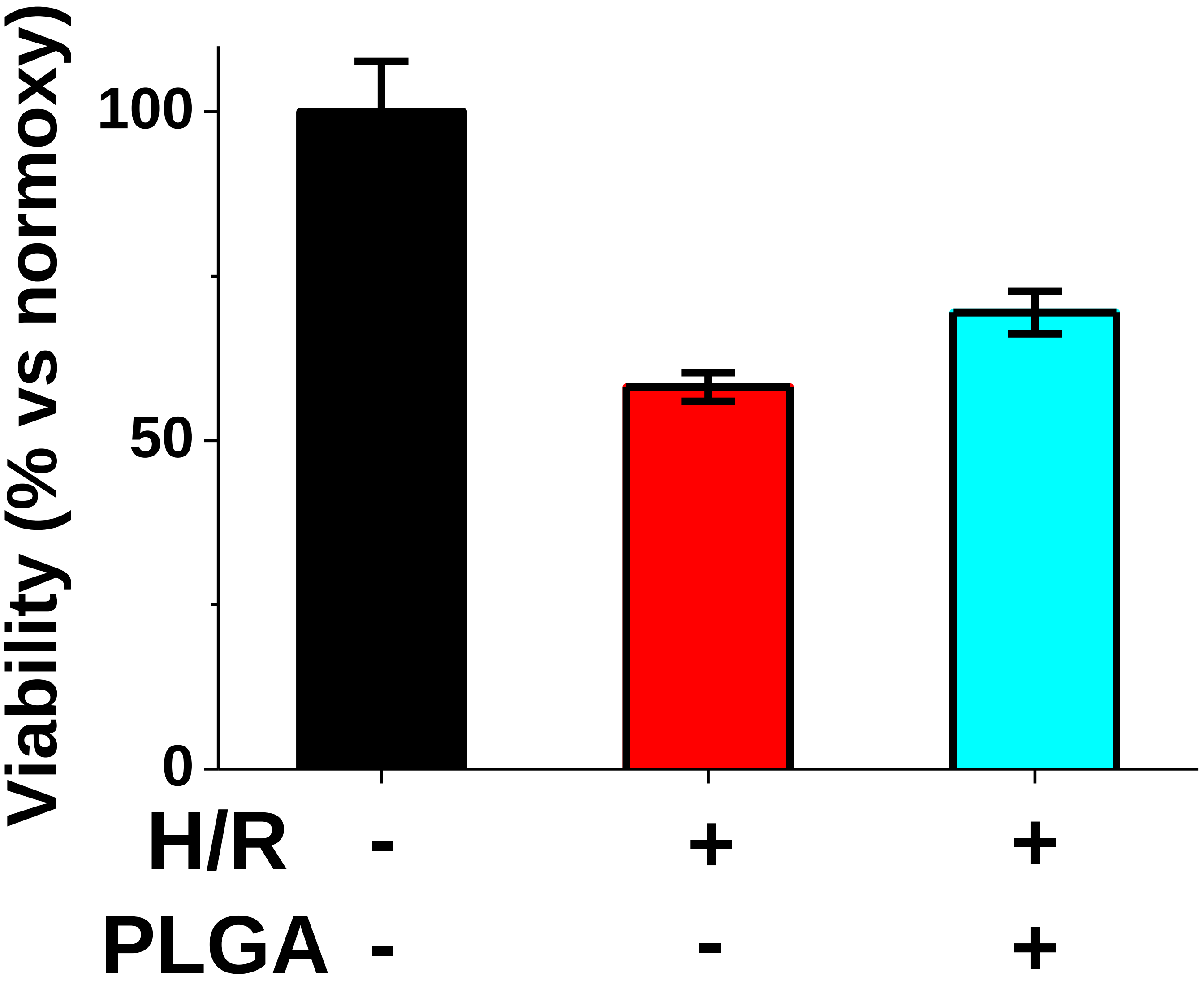

Supplementary Figure 6

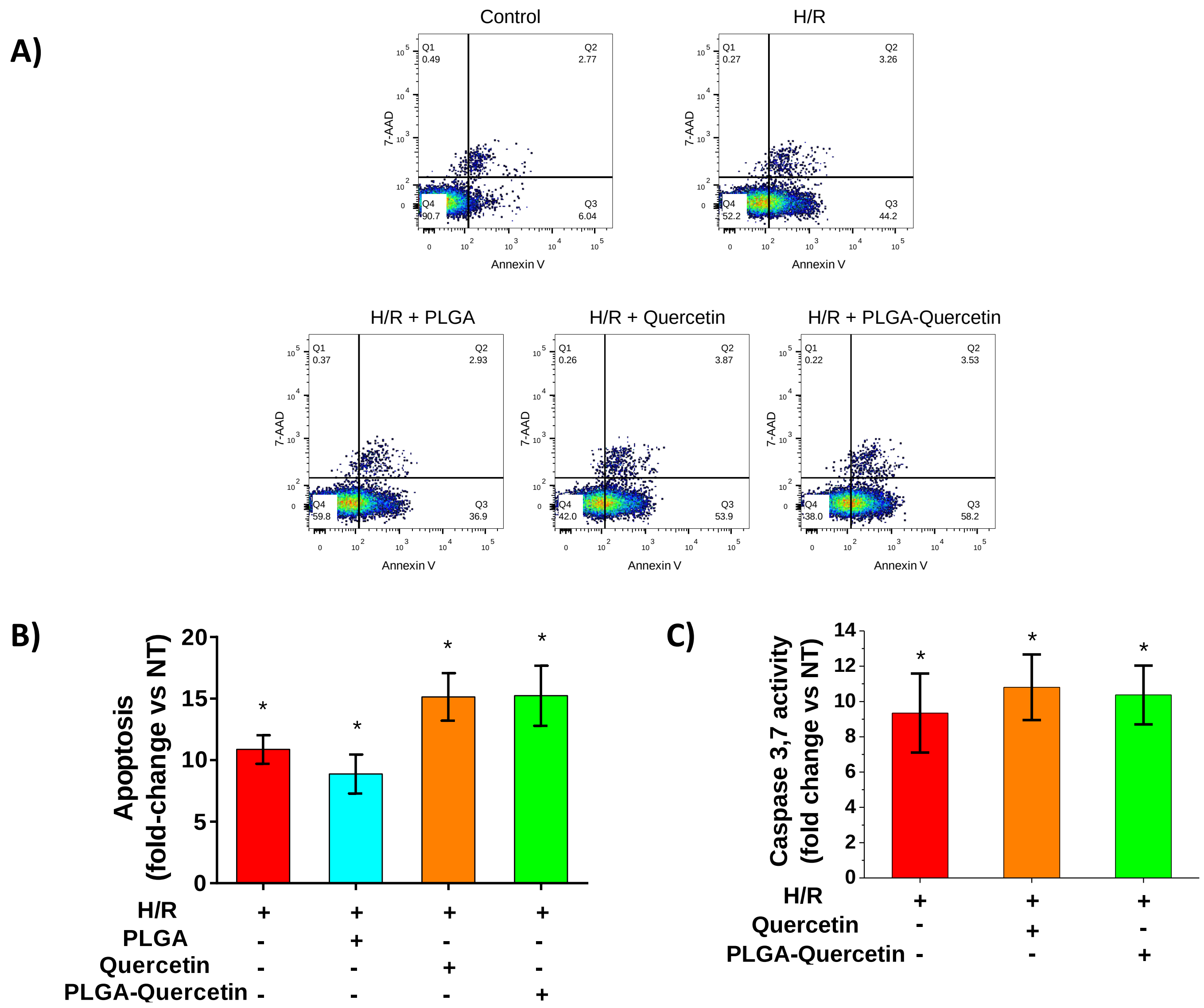

Supplement: Supplementary Materials — Supplementary Figure 1: representative TEM micrographs of (A) PLGA NPs and (B) PLGA-quercetin NPs. Scale bars of 100 nm. Insets: (A) representative TEM micrograph of PLGA NPs, scale bar of 200 nm; (B) representative TEM micrograph of PLGA-quercetin NPs, scale bar of 100 nm. Supplementary Figure 2: NP internalization into H9c2 cells after 24 h incubation. (A–C) Representative images of NT, FITC, and PLGA-FITC showing NPs can internalize into the cells. Merged images are composed of cell actin filaments (red), NP agglomerates (green), and nuclei (blue). Dotted yellow ellipses denote areas with NP accumulation. Scale bar of 40 μm. (D) NPs internalize as a function of the dose, and lines were fitted to equation (4). Statistical significance versus untreated (NT) cells, denoted as ∗, means P < 0.05. In (D), NT cells are denoted with 1e-3. (E) FITC fluorescence, at equimolar incubation doses, is higher when encapsulated in PLGA NPs. Supplementary Figure 3: ROS quenching of PLGA NPs in H9c2 cells due to antimycin A (AA): (A) mitochondrial O2 − and (B) H2O2. Supplementary Figure 4: encapsulated and free quercetin stability incubated in physiological solution, pH 7.4. (A) 380 nm/330 nm absorbance ratio as a function of time. Data were normalized against control groups at 0 h. ∗Statistical significance between each groups at different times vs. its control at 0 h. #Statistical significance between quercetin and PLGA-quercetin groups at specific times. (B) Selected absorbance spectra of encapsulated quercetin at 0, 48, and 168 h. (C) Selected absorbance spectra of free quercetin at 0, 48, and 168 h. Supplementary Figure 5: viability of H9c2 cells after 24 h of PLGA NP treatment, followed by the H/R. Supplementary Figure 6: apoptosis and necrosis of H9c2 cells after 24 h treatments, followed by H/R. (A) Representative dot plots of apoptosis and necrosis. (B) Apoptosis assessed by Annexin V. (C) Caspase 3/7 activity. [file 7683051.f1.pdf]
